# The Anisotropic Chemical Reaction Mechanism of 1,3,3-trinitroazetidine (TNAZ) under Different Shock Wave Directions by ReaxFF Reactive Molecular Dynamics Simulations

**DOI:** 10.3390/molecules27185773

**Published:** 2022-09-06

**Authors:** Junjian Li, Junying Wu, Yiping Shang, Muhammad Mudassar

**Affiliations:** State Key Laboratory of Explosion Science and Technology, Beijing Institute of Technology, Beijing 100081, China

**Keywords:** anisotropy, ReaxFF/lg, TNAZ, shock, molecular dynamics

## Abstract

1,3,3-Trinitroazetidine (TNAZ) has good thermal stability and low shock sensitivity, among other properties, and it has broad prospects in insensitive ammunition applications. In this study, a molecular dynamics calculation based on the ReaxFF-lg force field and multiscale shock technique (MSST) was used to simulate the shock-induced chemical reaction of TNAZ with different shock wave directions. The results showed that the shock sensitivity of TNAZ was in the order of [100] > [010] > [001]. There were significant differences in molecular arrangements in different shock directions, which affected the reaction rate and reaction path in different directions. The molecular arrangement in the [010] and [001] directions formed a “buffer” effect. The formation and cleavage of bonds, formation of small molecules and growth of clusters were analyzed to show the effect of the “buffer”. The polymerization reactions in the [010] and [001] directions appeared later than that in the [100] direction, and the cluster growth in the [010] and [001] directions was slower than that in the [100] direction. In different shock loading directions, the formation and cleavage mechanisms of the N-O bonds of the TNAZ molecules were different, which resulted in differences in the initial reaction path and reaction rate in the three directions

## 1. Introduction

1,3,3-Trinitroazetidine (TNAZ, C3H4N4O6) explosives, which have excellent performance, were first synthesized in 1984 by Archibald [1]. On one hand, TNAZ can form a eutectic mixture with other explosives and is expected to replace 2,4,6-trinitrotoluene (TNT) [1,2,3]. On the other hand, due to its excellent explosive properties, thermal safety performance and low sensitivity, TNAZ has broad prospects in the application of insensitive ammunition [2,3].

Reactive molecular dynamics simulations can be performed on systems of up to millions of atoms. Microscopic reaction information can be obtained at the atomic or molecular scale. Reaction molecular dynamics simulations have been widely used in the calculation of explosives such as 1,3,5-trinitro-1,3,5-triazacyclohex-ane (RDX), nitromethane (NM), 1,3,5,7-tetranitro-1,3,5,7-tetraazacyclooctane (HMX), and so on [4,5,6].

Many theoretical and experimental studies have been conducted on the thermal decomposition of TNAZ. Archibald obtained the unit cell parameters of TNAZ crystals by X-ray diffraction [1]. Anderson used an STMBMS mass spectrometer to study the thermal decomposition of TNAZ. The results showed that the main initial decomposition products of TNAZ were NO_2_ and NO, and the breakage of the N-NO_2_ and C-NO_2_ bonds was the main path of the initial thermal decomposition of TNAZ [7]. Olah also obtained similar conclusions by mass spectrometry [8]. Behrens studied the process of bond cleavage by simultaneous thermogravimetric modulated beam mass spectrometer. They found that the cleavage of the N-NO_2_ group preceded that of the C-NO_2_ cleavage in thermal decomposition of TNAZ [9]. Agrawal studied the melting point and isothermal compression properties of defect-free TNAZ crystals by molecular simulation. The unit cell parameters and thermodynamic melting point of TNAZ crystals obtained with the AMBERSTR force field were basically consistent with the experimental values [8]. Although TNAZ explosives have been the subject of some studies and have been used in some applications, few studies on shock reactions mechanism have been conducted to date, and no studies have been reported from the atomic and molecular microscopic levels. The mechanism and characteristics of the shock reactions of TNAZ explosives are not well understood. The reaction mechanism of TNAZ under shock conditions, which is studied using molecule dynamics, is of great significance to the shock response performance and safety evaluation of TNAZ explosives.

The multiscale shock technique (MSST) was proposed by Reed in 2003 [10]. Through the method, researchers can accurately obtain the evolution process of various thermodynamic parameters, which greatly reduces the computational cost. The MSST is an effective means to study the reaction of materials under shock [11,12,13,14,15,16,17,18,19,20,21]. In 2009, Manaa et al. used the MSST to study the shock response of 1,3,5-triamino-2,4,6-trinitrobenzene (TATB) crystals under different shock wave velocity loadings; they found that the formation of many clusters during the shock caused the low shock sensitivity of TATB [22]. In 2019, Liu et al. used the MSST with the ReaxFF molecular dynamics calculation method to study the initial reaction mechanism of ε-CL-20 crystals under shock. They found that the detonation reaction occurred when the cleavage rates of C-N and C-H were greater than 3.11 and 4.15%/ps, respectively [23]. In 2019, Liu et al. studied the initial decomposition mechanism of energetic cocrystal 2,4,6,8,10,12-hexantro-2,4,6,8,10,12-hexazaiso-wurzitane (CL-20)/1,3,5,7-tetranitro-1,3,5,7-tetrazacy-clooctane (HMX) under a steady shock wave. They found that after the shock wave application, the energetic cocrystals successively underwent an induction period, fast compression, slow compression and expansion processes. The fast and slow compression processes corresponded to the fast and slow decomposition of the reactants, respectively [24]. In 2019, Liu et al. studied ReaxFF molecular dynamics simulations of the shock-induced reaction initiation in TNT. They found that the formation of TNT dimers and decomposition to C7H5O5N3 were the dominant initial routes for shock-induced reaction initiation. In addition, they also analyzed the formation and evolution of carbon clusters [25]. In 2020, Huang et al. studied anisotropic hydrogen bond structures and the orientation dependence of shock sensitivity in 1,3,5-tri-amino-2,4,6-tri-nitrobenzene (TATB). They found that the temperature, stress, volume compressibility and decomposition rate of TATB were strongly dependent on the shockwave direction [26]. These studies show that by using the MSST method, researchers can explain the macroscopic phenomena at the atomic and molecular levels and obtain detailed physical properties (pressure and temperature) of the supercell under shock.

The results of previous studies did not discuss the reaction mechanism from the perspective of the system structure after shock compression. This paper discusses the similarities and differences between the reactions in various directions after shock from the perspective of the compressed crystal structure and intermolecular interaction. The shock in different directions leads to differences in the crystal structure and molecular structure. These differences lead to differences in the physical properties of supercells, which affect the subsequent chemical reaction pathways and products. In this paper, ReaxFF-lg [27,28] and MSST are used to study the molecular dynamics of the TNAZ shock reaction process. TNAZ in the [100], [010] and [001] directions at velocities of 8, 9, 10 and 11 km∙s^−1^ is studied. TNAZ supercells at different shock loading rates are examined. The particle velocity, system pressure, shock Hugoniot relationship, the effect of the shock loading velocity on the reaction path of the explosive and the bond information are analyzed.

## 2. Computational Methods

**Figure 1:**
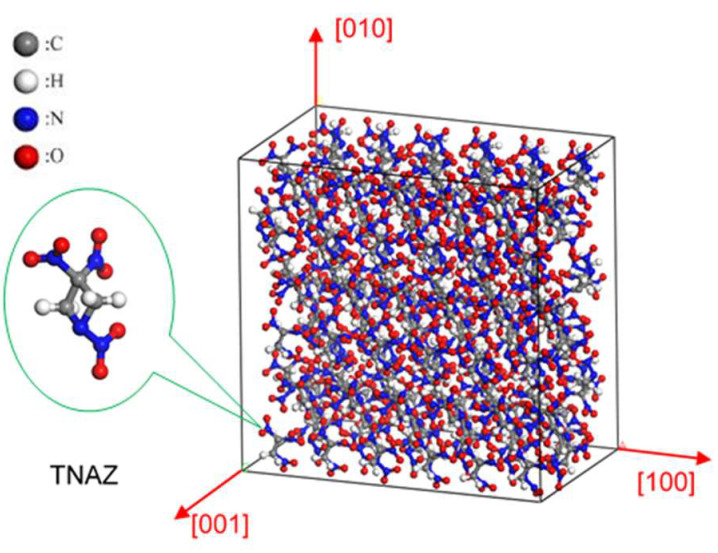
Schematic diagram of the TNAZ molecular structure.

The simulations were performed with LAMMPS using ReaxFF-lg and MSST. The single-crystal structure of TNAZ was obtained from the X-ray diffraction crystal data [1]. The space group of TNAZ is Pbca and a unit cell contains 8 molecules. Then, it was expanded along the a, b and c directions to construct a 6 × 3 × 1 TNAZ supercell. The 6 × 3 × 1 TNAZ supercell was constructed in Material Studio (MS) software and contained 144 molecules (2448 atoms). The parameters of the expanded supercell were a = 34.398 Å, b = 33.381 Å, c = 21.496 Å, α = 90.0°, β = 90.0° and γ = 90.0°. A schematic diagram of the TNAZ molecule and supercell is shown in Figure 1. Before shock loading, the supercell was relaxed to obtain a stable structure under normal temperature and pressure. The structure was first relaxed by energy minimization, followed by thermalization at 298 K. A molecular dynamics (MD) simulation was first performed for 10 ps with the canonical ensemble (NVT, constant number of particles, volume and temperature) using the Berendsen thermostat. Then, a pressure relaxation at 0 GPa was performed for 5 ps using the isobaric–isothermal ensemble (NPT, constant number of particles, pressure and temperature) with the Nosé–Hoover thermostat and barostat. In the relaxation and formal calculations, the time step was 0.05 fs and the coupling coefficient was 100. We considered the use of different cut-off radii for different atom pairs proposed by reviewers. Here, different key types were set to different cut-off radii [14] (as shown in Table S1, and cut-off* is used in the following text). Finally, the crystal equilibrium structure at room temperature was obtained, and the density of the equilibrium structure was 1.84 g·cm^−3^. Huang et al. verified the applicability of ReaxFF-lg to TNAZ, which is not discussed here [29]. After obtaining the crystal equilibrium structure, the TNAZ supercell system was subjected to shock in the [100], [010] and [001] directions. The shock wave velocities were 8, 9, 10 and 11 km∙s^−1^.

## 3. Results and Discussion

### 3.1. Shock Wave Velocity and Particle Velocity

The selected particle velocity is the particle velocity at the moment when the system yields, which is used to describe the state of the system after shock compression. During the shock process, the shock velocity has a linear relationship with the postwave particle velocity, i.e., the Hugoniot relationship. The formula is

(1) $$u_{s}=C+Su_{p}$$

where *C* is the sound velocity of the dielectric material, and *S* is the adiabatic bulk modulus.

**Figure 2:**
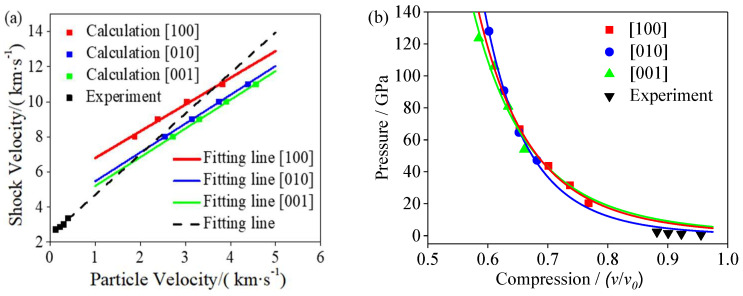
(**a**) Relationship between TNAZ shock velocity and particle velocity. (**b**) Relationship between pressure and specific volume.

The relationship between the TNAZ shock velocity and particle velocity and the relationship between the pressure and specific volume were obtained through calculations. As shown in Figure 2, the calculated data were consistent with the experimental results, which indicated that the MSST could accurately evaluate the TNAZ state under the shock wave [30].

### 3.2. Temperature and Pressure in Different Shock Directions

**Figure 3:**
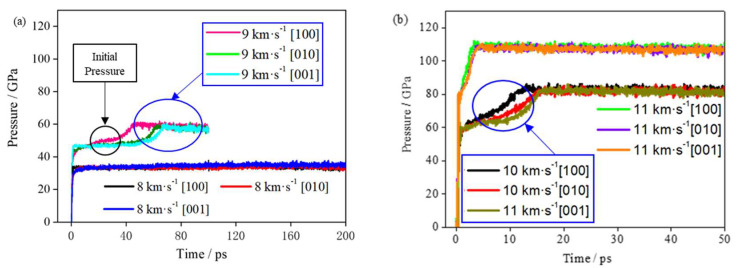
(**a**) Evolution of pressure at 8 and 9 km∙s^−1^. (**b**) Evolution of pressure at 10 and 11 km∙s^−1^.

The total energy curves of the TNAZ system under different shock wave loading conditions are shown in Figure S1. Figure 3 shows the hydrostatic pressure curve of the system with time in different shock velocities. At the initial moment, when the system is subjected to shock compression, the pressure quickly jumps. With a greater shock wave velocity, a larger initial shock wave pressure is generated, and the pressurization time is shorter. Then, the reaction begins to occur, and the pressure further rapidly increases. The rate of the pressure increase and its peak value are related to the shock wave velocity and direction. When the shock wave velocities are identical, the pressures in different loading directions have a similar evolution trend. A greater shock wave velocity corresponds to a greater pressure of the system.

**Figure 4:**
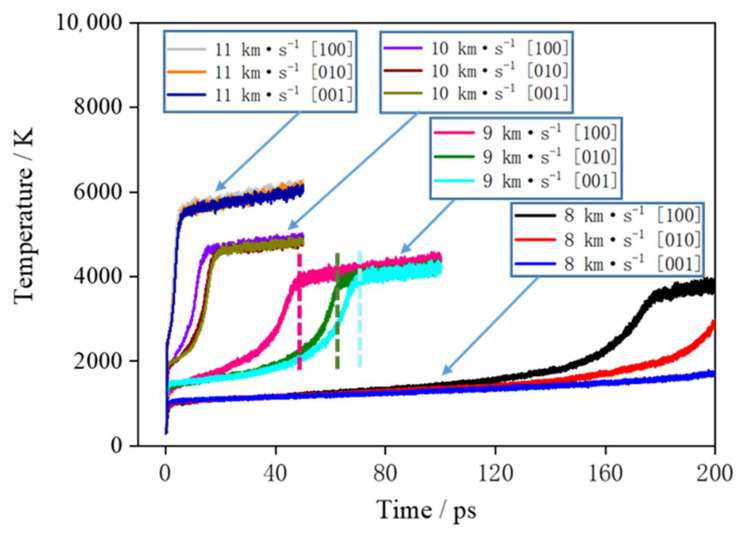
Evolution of temperature at different shock velocities. Taking the TNAZ supercell at 9 km∙s^−1^ as an example, the stage from 0 ps to the position of the dotted line is the rapid reaction stage. The stage from the position of the dotted line to 100 ps is the slow reaction stage.

Table S2 shows the initial pressure value, stable pressure value and corresponding time in different directions. The initial pressure in this paper refers to the first peak pressure when the shock wave was applied to the supercell. When the shock velocity is 8 km∙s^−1^, the initial shock pressures are 33.55, 33.31 and 33.60 GPa in the [100], [010] and [001] directions, respectively. At a shock velocity of 8 km∙s^−1^, the reaction is slow, and the pressure of the TNAZ system does not significantly increase within 150 ps. According to the slope of the curve in Figure 4, the chemical reaction is divided into the fast chemical reaction and the slow chemical reaction stage. Therefore, no statistical analysis was performed at 8 km∙s^−1^. When the shock velocity is greater than or equal to 9 km∙s^−1^, the pressure of the TNAZ system obviously increases and reaches a stable value. In Table S2, when the shock velocity is identical, as the reaction progresses, the rate of pressure increase in the [100] direction is faster than that in the [010] and [001] directions. The rate of chemical reactions in the [100] direction is faster than that in the [010] and [001] directions. This result also indicates that TNAZ has a strong anisotropy in the pressure under shock.

Figure 4 shows the curve of the supercell temperature with time. First, the supercell is subjected to shock compression under shock, and kinetic energy is converted into internal energy. Behind the shock wave front, the temperature of the medium sharply increases; then, rapid chemical reactions occur, which release a large amount of heat. Here, the stage where the temperature rapidly increases is called the rapid chemical reaction stage, and the stage where temperature slowly increases is called the slow reaction stage. When the rapid chemical reactions are completed, there are many clusters in the products, and the clusters continue to decompose and produce the final small-molecule products. There are relatively slow chemical reactions in this process, and the released heat is much less than that released in the rapid chemical reaction stage. When the shock velocity is 8 km∙s^−1^, due to the low compression intensity of the shock, the rapid chemical reactions of different directions begin at 130 ps. When the shock velocity is 9 km∙s^−1^, the rapid chemical reactions of different directions end in 50–70 ps and enter the slow chemical reaction stage. When the shock velocity is 10 and 11 km∙s^−1^, the rapid chemical reactions of different directions are completed in 0–20 ps, and the slow chemical reaction stage begins.

At shock velocities of 8, 9, 10 and 11 km∙s^−1^, the temperatures in the [100] direction are 1243, 3987, 4903 and 6106 K at 50 ps, respectively. When the shock velocity is larger, the temperature of the system rises faster, the system starts to react quickly, the reaction rate is larger, and the system enters into the slow chemical reaction stage earlier. From the temperature curves of 9 and 10 km∙s^−1^, it can be seen that in the rapid chemical reaction stage, the temperature rise rate of the system in the [100] direction is significantly higher than that of the [010] and [001] directions. It shows that the rate of rapid chemical reaction of TNAZ under the shock loading in the [100] direction is higher than that of the other two directions. When the shock wave velocity is 8 km∙s^−1^, due to the low shock pressure, the chemical reaction is slow, and within the calculation time of 200 ps, only a small increase in the temperature of the system occurs in the [100] direction. For the shock wave velocity of 11 km∙s^−1^, due to the large shock loading intensity, the explosive completes a rapid chemical reaction in a very short time, and the temperature changes of the system are not significantly different under different shock loading directions. In conclusion, it can be seen from Figure 3 and Figure 4 that the TNAZ exhibits obvious anisotropy characteristics for shock wave loading with different directions, and the difference is the most obvious for the velocity of 9 km∙s^−1^.

### 3.3. Study on the Causes of TNAZ Anisotropy

TNAZ has strong anisotropic pressure and temperature responses in different directions, which may be related to the molecular arrangements in different directions. Here, this paper takes the supercell with a shock velocity of 9 km∙s^−1^ as an example for the analysis.

**Figure 5:**
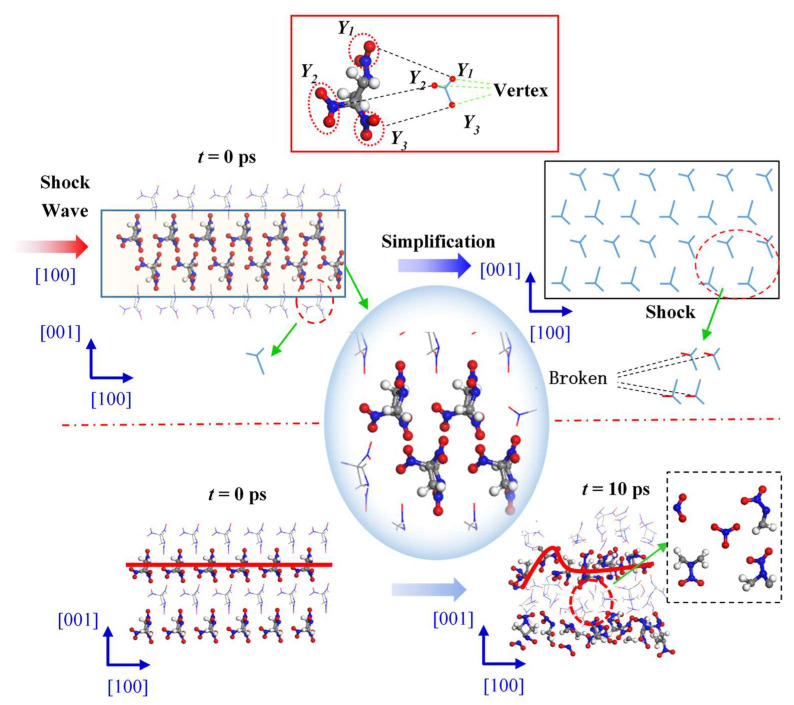
Molecular arrangement of TNAZ in the [100] direction.

Figure 5 shows the changes in internal structure of the TNAZ supercell shocked in the [100] direction. For the convenience of observation, this paper uses the “Y” figure to represent the approximate shape of the TNAZ single molecule in the [100] direction. From the perspective of molecular arrangement, TNAZ molecules are arranged in a straight line in the [100] direction, and the direction of the molecules on the same line is identical. When the supercell is shocked in the [100] direction, a vertex of the “Y” shape is squeezed with the adjacent “Y” shape. The squeezing easily breaks the molecular structure and makes the supercell react. As shown in Table 1, when t = 0 ps, the initial number of TNAZ molecules is 144. When t = 10 ps, the number of TNAZ molecules is reduced to 94 in the [100] direction. Compared to the [010] and [001] directions, the [100] direction has more small molecules. There are five NO_2_ molecules, two HNO_2_ molecules, one O_2_ molecule and two NO_3_ molecules, respectively. Along the [100] direction, the arrangement of TNAZ molecules cannot be maintained, and the molecular structure has been significantly deformed. Thus, in this direction, the reaction rate of the supercell is the fastest.

**Figure 6:**
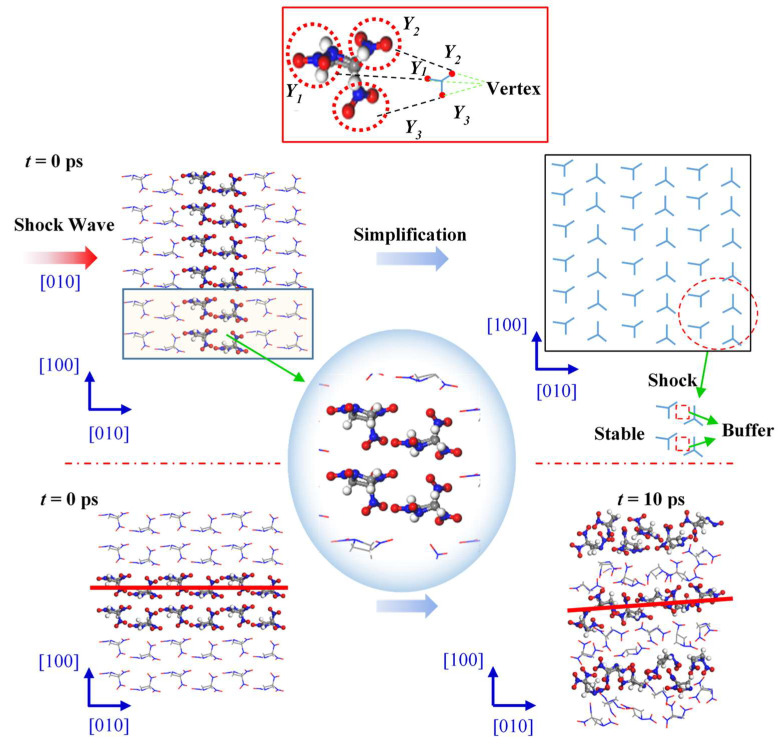
Molecular arrangement of TNAZ in the [010] direction.

Figure 6 shows changes in the internal structure of the TNAZ supercell shocked in the [010] direction. From the perspective of the molecular arrangement, the TNAZ molecules are also arranged in a straight line in the [010] direction. However, the directions of adjacent TNAZ molecules on the same line are opposite. When the supercell is shocked in the [010] direction, a vertex of the “Y” shape (Y1) and two vertices of the adjacent “Y” shape (Y2 and Y3) are near each other. In the [010] direction, there is a large space between adjacent TNAZ molecules to form a “buffer” for the shock in this direction. The supercell remains relative stable in this direction. As shown in Table 2, when t = 0 ps, the initial number of TNAZ molecules is 144. When t = 10 ps, the TNAZ molecules are shocked in the [010] direction, and the number of TNAZ molecules is reduced to 121. The TNAZ molecules maintain a linear arrangement in the [010] direction, and the molecular structure remains intact. At 10 ps, the number of unreacted TNAZ molecules in the system after being shocked in the [100] direction is less than that in the [100] direction. There are not many small molecules in the [010] direction. The number of NO_2_, HNO_2_, O_2_ and NO_3_ is two, zero, one and two, respectively. Thus, in this direction, the rate of reactions is lower than in the [100] direction.

**Figure 7:**
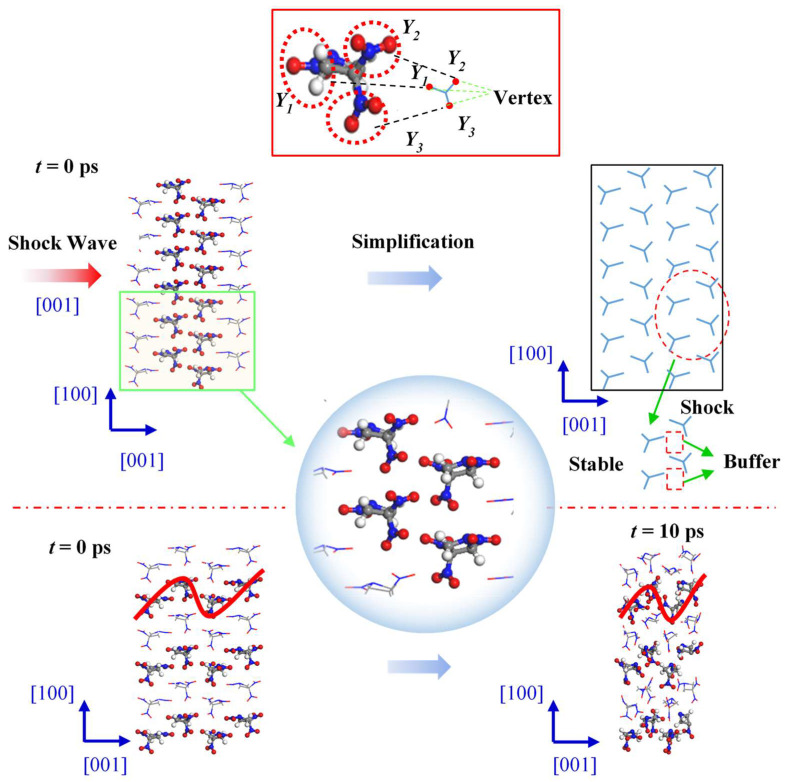
Molecular arrangement of TNAZ in the [001] direction.

Figure 7 shows the changes in internal structure of the TNAZ supercell shocked in the [001] direction. From the perspective of the molecular arrangement, the TNAZ molecules are arranged as a wavelike line in the [001] direction. When the supercell is shocked in the [001] direction, a vertex of the “Y” shape (Y1) is also near two vertices of the adjacent “Y” shape (Y2 and Y3). When adjacent TNAZ molecules are close, there is a large space between the molecules, which form a type of “buffer” for the shock in this direction. Thus, the molecular structure is not easily destroyed in the [001] direction, and the supercell is stable in this direction. As shown in Table 3, when t = 0 ps, the initial number of TNAZ molecules is also 144. When t = 10 ps, the number of TNAZ molecules is reduced to 132 in the [001] direction. The TNAZ molecules maintain the arrangement of a wavelike line in the [001] direction, and many molecules are intact. There are not many small molecules in the [010] direction. The number of NO_2_, HNO_2_, O_2_ and NO_3_ is four, zero, one and two, respectively. Hence, in this direction, the rate of reactions is also lower than that in the [100] direction.

Based on the steric hindrance model proposed by Dick et al. [31], during the impact sliding process, molecules avoid close contact between each other through rotational deformation and other methods to cushion the impact compression. In the [100] direction, the molecules are arranged in a good order, the front-row molecules directly collide with the back-row molecules, and the impact compression effect cannot be buffered. In the [010] and [001] directions, due to the staggered arrangement of molecules, along the compression direction, there are some slip spaces between the molecules, so the molecules move into the intermolecular space, rotate and deform to avoid molecular collisions. Therefore, the impact sensitivity of TNAZ in the [100] direction is higher than that in the other two directions.

### 3.4. Initial Reactions and the Chemical Bonds

The high-frequency reactions of the first 10 ps at 9 km∙s^−1^ is shown in Table 4. Bimolecular polymerization first occurs in the shock. By the time of bimolecular polymerization in different directions, the “buffering” effect in different directions can be revealed. In the [100], [010] and [001] directions, the time of bimolecular polymerization is 0.40 ps, 0.975 ps and 1.375 ps, respectively. Compared to the [100] and [001] directions, the molecular arrangement along the [010] direction can significantly increase the “buffering” effect. The polymerization reaction is exothermic, and the pyrolysis reaction is endothermic. Therefore, the frequencies of the net polymerization reactions in the three directions can be analyzed, i.e., the frequency of the polymerization reactions minus the frequency of the pyrolysis reactions. In the [100], [010] and [001] directions, the frequencies of the net polymerization reactions are 16, 4 and 1, respectively. The reactions in the [100] direction release the most heat, and the temperature increase of the TNAZ supercell is the fastest; the reactions in the [001] direction releases the least heat, and the temperature increase of the TNAZ supercell is the slowest. A high temperature can accelerate the unimolecular reactions of TNAZ. In terms of reaction time, the unimolecular reactions in the [100] direction take place earlier than that in the [010] and [001] directions. Above all, the sensitivity order of TNAZ in different directions is [100] > [010] > [001].

**Figure 8:**
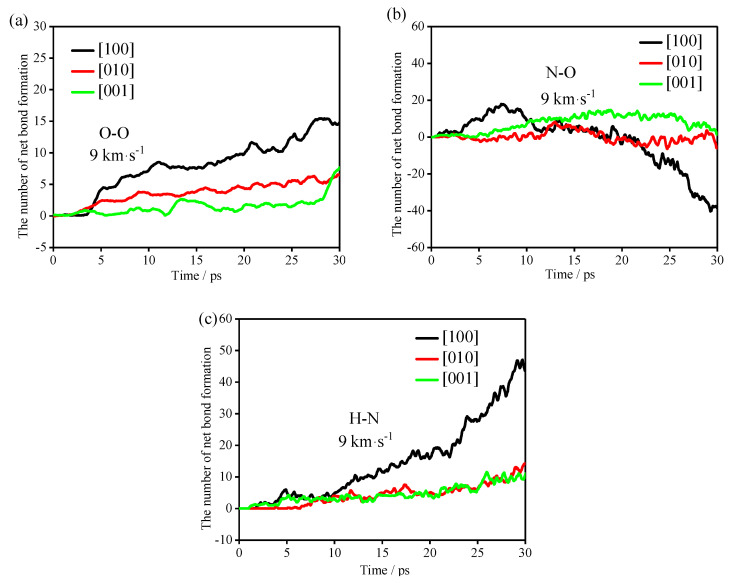
Net bonding of (**a**) O-O bonds, (**b**) N-O bonds and (**c**) H-N bonds.

To study the formation of clusters in the early stage of the reactions, the bonds (O-O, N-O and H-N) with obvious changes in the early stage of the reactions were analyzed, as shown in Figure 8. In Figure 8, the positive value plotted on the *Y*-axis represents the number of positive net bonds, and the negative value represents the number of negative net bonds, that is, the number of net broken bonds.

It can be seen from Table 4 that the polymerization of the TNAZ system frequently occurs before 10 ps. Therefore, in the first 10 ps reactions, the TNAZ molecules in the [100] shock direction are mainly combined into clusters by the formation of O-O, N-O and H-N bonds. The TNAZ molecules in the [010] shock direction are mainly combined into clusters by the formation of H-N bonds. The TNAZ molecules in the [001] shock direction are mainly combined into clusters by the formation of O-O bonds. In the formation of O-O and H-N bonds, the net bonding rate is the fastest in the [100] shock direction. Although the net bonding of O-O and H-N bonds in the [010] and [001] shock directions also show differences, the difference is not significant. Compared with the change trend of O-O and H-N bonds in the three directions, the change trend of the N-O bonds in the three directions is obviously different. In the [100] shock direction, the N-O bonds form first and then break. In the [010] and [001] shock directions, the trend of net bond formation is not obvious, and almost all of them show net bond breaking. This phenomenon may be related to the shock direction. In the [100] direction, the number of N-O bonds has an upward peak during the first 5–10 ps. This is due to the close distance between the N and O atoms in adjacent TNAZ molecules in the [100] direction. Shock wave compression makes the distance between the N and O atoms shorter than the cut-off radius, resulting in an increase in the number of N-O bonds.

The occurrence of the chemical reaction must be accompanied by the breaking of bonds. The shock directions affect the formation and cleavage of bonds at the initial stage. In other words, the shock directions affect the initial reaction path of the TNAZ system.

### 3.5. Main Intermediate Products and Cluster Analysis

In different shock directions, the small-molecule products of TNAZ are H_2_O, N_2_, CO_2_, NO_2_, O_2_, NO_3_, HNO_2_, etc. Here, typical small-molecule products such as O_2_, NO_3_ and HNO_2_ were selected for the analysis. When the shock velocities are identical, the mechanisms of reactions in different directions to form small molecules are not exactly identical. When the supercell is shocked in a certain direction, the distance between the atoms in the direction of the shock decreases. When the atoms or groups on some molecules are detached, the decrease in distance makes it easy for these atoms or groups to combine, which causes the differences between the reaction paths of the small molecules in subsequent reactions.

Table 5 shows the high-frequency reactions of the O_2_ formation in three directions. The net response frequencies in the [010] direction, [001] direction and [100] direction are −1, 13 and 6, respectively. From the reactants, the net reaction frequency at which O_2_ molecule is produced via single-molecule decomposition is the highest in the [010] direction with the value of 12. Most of the reactants in the [001] directions are polymers. 

Thus, most of the TNAZ molecules in the [001] directions first polymerize into polymers; then, these polymers decompose into O_2_ molecules. In the [001] directions, O_2_ is mainly produced from the polymer, while in the [010] direction, O_2_ is mainly formed by the decomposition of TNAZ molecules. In terms of reaction time, the time sequence of O_2_ appearance in each direction is different. The appearance time of O_2_ in the [100], [010] and [001] directions is 6.70, 3.13 and 13.15 ps, respectively. Thus, in the [010] direction, O_2_ molecules appear first.

Table 6 shows the high-frequency reactions of NO_3_ formation in three directions. Compared to O_2_ molecules, most of the reactants are TNAZ molecules in the reactions, and NO_3_ generally appears earlier than O_2_. In the [100] direction, the net reaction frequency of NO_3_ formation is 9; in the [010] direction, the net frequency is 16; finally, in the [001] direction, the net frequency is 17. The [100] direction produces fewer NO_3_ molecules than that of the other two directions. When the supercell is shocked in the [001] direction, some reactants are polymers, which are not observed in the cases of the other two directions. In terms of reaction time, the time sequence of NO_3_ appearance in each direction is different. In the case of shock wave loading in the [100] direction, NO_3_ molecules first appear at 1.73 ps.

Table 7 shows the high-frequency reactions of HNO_2_ formation in three directions. Compared to O_2_ and NO_3_ molecules, the single-molecule reactants have higher reaction frequency. In terms of reactants, the reaction frequency at which reactants are polymers is 0 in the three directions, which is obviously different from the O_2_ and NO_3_ molecules. Compared to the other two directions, more HNO_2_ molecules are produced when shocked under the shock wave loading in the [001] direction. In terms of reaction time, HNO_2_ appears in the [100], [010] and [001] directions at 4.70 ps, 11.75 ps and 2.55 ps, respectively. Thus, in the [001] direction, HNO_2_ molecules appear first.

In conclusion, O_2_, NO_3_ and HNO_2_ first appear in the [010], [100] and [001] directions, respectively. The appearance time of small molecules is affected by the directions of shock and not directly related to the sensitivity. The reaction paths of small molecules in three directions are also different. The reactants of O_2_ and NO_3_ are many polymers in the [001] directions but less in the [100] and [010] direction. Compared with O_2_ and NO_3_, the reactants of HNO_2_ are almost single molecules, and the reaction mainly occurs in the [001] direction.

**Figure 9:**
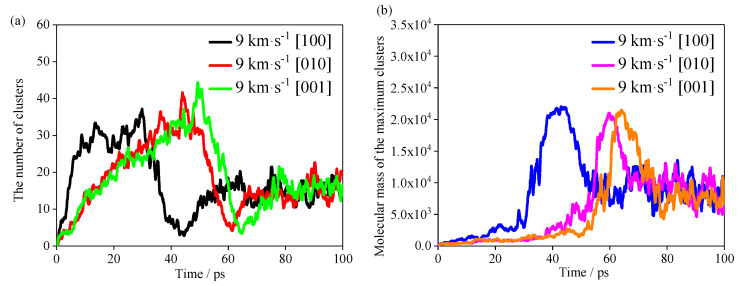
(**a**) Evolution of the number of the clusters. (**b**) Molecular mass of the maximum clusters.

The evolutions of the number of clusters and molecular mass of maximum clusters in the TNAZ system with different shocks are shown in Figure 9a,b. For the convenience of statistics and analysis, “clusters” refers to molecules whose relative molecular weight is more than that of a single TNAZ molecule. First, the most rapid increase in number of clusters occurs in the [100] direction, but the most rapid decrease in number of clusters also occurs in this direction. The numbers of clusters in the [010] and [001] directions reach the maximum value at very similar times, but they are later than that in the [100] direction. When the number of clusters decreases, the molecular weight of the largest cluster rapidly increases, which indicates that the reduced clusters are aggregated to the largest cluster. The shock in the [100] direction makes TNAZ molecules form large clusters with a molecular mass above 20,000. Under the shock of the [100] direction, the time it takes for the number of clusters to reach 30 is approximately 20 ps, and the molecular mass of the largest cluster rapidly increases, so more TNAZ molecules participate in the polymerization reactions in the [100] direction. After approximately 45 ps, the cluster with the largest molecular mass begins to decompose, and those in the [100] direction decompose before those in the [010] and [001] directions. In summary, there is a hysteresis in the response of the [010] and [001] directions to the pressure, and the arrangement of molecules in this direction can buffer the pressure, so that the shock sensitivity of TNAZ supercell is lower in the [010] and [001] directions than in the [100] direction.

## 4. Conclusions

In this paper, the ReaxFF-lg force field and MSST method were used to study the anisotropic reaction mechanism of the TNAZ shock compression process. The chemical reactions in different shock directions were calculated. When the shock velocities were identical, the increasing rates of pressure and temperature in the [100] direction were greater than those in the [010] and [001] directions. There were significant differences in molecular arrangements between different directions, which affected the chemical reaction rate. Due to the differences in arrangements, the shock sensitivity of the TNAZ supercells in different directions was ranked as [100] > [010] > [001]. The shock directions affected the formation and cleavage of the N-O bonds in the initial stage. In other words, the shock directions affected the initial reaction path of the TNAZ system. The shock directions also affected the reaction paths and appearance time of small molecules such as O_2_, NO_3_ and HNO_2_. According to the reactions of the first 10 ps and the evolution of the clusters, the polymerization reactions in the [010] and [001] directions appeared later than that in the [100] direction, and the cluster growth in the [010] and [001] directions was slower than that in the [100] direction. Therefore, the TNAZ molecules in the [010] and [001] directions can form a buffer for the shock wave. This paper provides not only more information about the properties of explosives for other scholars studying TNAZ explosives but also new ideas and methods for studying the anisotropic reaction principle of energetic materials.

## Figures and Tables

**Table 1:** Number of TNAZ molecules and other small molecules in the [100] direction.

Time (ps)	C_3_H_4_N_4_O_6_ (TNAZ)	NO_2_	HNO_2_	O_2_	NO_3_
0	144	0	0	0	0
10	94	5	2	1	2

**Table 2:** Number of TNAZ molecules and other small molecules in the [010] direction.

Time (ps)	C_3_H_4_N_4_O_6_ (TNAZ)	NO_2_	HNO_2_	O_2_	NO_3_
0	144	0	0	0	0
10	121	2	0	1	2

**Table 3:** Number of TNAZ molecules and other small molecules in the [001] direction.

Time (ps)	C_3_H_4_N_4_O_6_ (TNAZ)	NO_2_	HNO_2_	O_2_	NO_3_
0	144	0	0	0	0
10	132	4	0	1	2

**Table 4:** Reactions of TNAZ in the first 10 ps.

Shock Velocity and Directions (km∙s^−1^)	Net Reaction Frequency	Frequency	Reaction Time (ps)	Reaction
9 [100]	12	58	0.40–9.825	2C_3_H_4_N_4_O_6_ → C_6_H_8_N_8_O_12_
		46	0.425–9.85	C_6_H_8_N_8_O_12_ →2C_3_H_4_N_4_O_6_
	4	39	2.15–9.975	C_3_H_4_N_3_O_4_ + C_3_H_4_N_4_O_6_ → C_6_H_8_N_7_O_10_
		35	2.40–9.90	C_6_H_8_N_7_O_10_ → C_3_H_4_N_3_O_4_ + C_3_H_4_N_4_O_6_
	18	38	0.075–9.40	C_3_H_4_N_4_O_6_ → C_3_H_4_N_3_O_4_ + NO_2_
		20	0.60–9.425	C_3_H_4_N_3_O_4_ + NO_2_ → C_3_H_4_N_4_O_6_
9 [010]	3	49	0.975–9.675	2C_3_H_4_N_4_O_6_ → C_6_H_8_N_8_O_12_
		46	1.0–9.775	C_6_H_8_N_8_O_12_ → 2C_3_H_4_N_4_O_6_
	1	23	1.275–9.975	C_3_H_4_N_3_O_4_ + C_3_H_4_N_4_O_6_ → C_6_H_8_N_7_O_10_
		22	1.575–9.95	C_6_H_8_N_7_O_10_ → C_3_H_4_N_3_O_4_ + C_3_H_4_N_4_O_6_
	5	19	0.95–9.9	C_3_H_4_N_4_O_6_ → C_3_H_4_N_3_O_4_ + NO_2_
		14	0.975–9.925	C_3_H_4_N_3_O_4_ + NO_2_ → C_3_H_4_N_4_O_6_
9 [001]	−1	53	1.375–9.875	2C_3_H_4_N_4_O_6_ → C_6_H_8_N_8_O_12_
		54	0.525–9.925	C_6_H_8_N_8_O_12_ → 2C_3_H_4_N_4_O_6_
	2	30	1.525–9.90	C_3_H_4_N_3_O_4_ + C_3_H_4_N_4_O_6_ → C_6_H_8_N_7_O_10_
		28	1.575–9.925	C_6_H_8_N_7_O_10_ → C_3_H_4_N_3_O_4_ + C_3_H_4_N_4_O_6_
	4	34	1.15–9.95	C_3_H_4_N_4_O_6_ → C_3_H_4_N_3_O_4_ + NO_2_
		30	2.40–9.75	C_3_H_4_N_3_O_4_ + NO_2_ → C_3_H_4_N_4_O_6_

**Table 5:** Reactions of O_2_ molecule production.

Shock Velocity and Directions (km∙s^−1^)	Net Reaction Frequency	Frequency	Reaction Time (ps)	Reaction
9 [100]	0	23	6.70–24.63	C_3_H_4_N_4_O_7_ → C_3_H_4_N_4_O_5_ + O_2_
		23	6.65–24.63	C_3_H_4_N_4_O_5_ + O_2_ → C_3_H_4_N_4_O_7_
	−2	6	21.83–23.40	C_9_H_13_N_13_O_18_ → C_9_H_13_N_13_O_16_ + O_2_
		8	21.75–23.20	C_9_H_13_N_13_O_16_ + O_2_ → C_9_H_13_N_13_O_18_
	0	4	20.93–22.38	C_6_H_7_N_8_O_11_ → C_6_H_7_N_8_O_9_ + O_2_
		4	20.95–22.88	C_6_H_7_N_8_O_9_ + O_2_ → C_6_H_7_N_8_O_11_
	1	4	11.35–13.00	C_6_H_9_N_8_O_11_ → C_6_H_9_N_8_O_9_ + O_2_
		3	11.37–12.98	C_6_H_9_N_8_O_9_ + O_2_→ C_6_H_9_N_8_O_11_
9 [010]	4	118	3.13–50.45	C_3_H_4_N_4_O_7_ → C_3_H_4_N_4_O_5_ + O_2_
		114	4.50–40.08	C_3_H_4_N_4_O_5_ + O_2_ → C_3_H_4_N_4_O_7_
	4	16	43.65–50.45	C_3_H_4_N_4_O_5_ → C_3_H_4_N_4_O_3_ + O_2_
		12	44.05–49.23	C_3_H_4_N_4_O_3_ + O_2_ → C_3_H_4_N_4_O_5_
	4	13	18.53–39.33	C_3_H_4_N_3_O_6_ → C_3_H_4_N_3_O_4_ + O_2_
		17	18.53–39.33	C_3_H_4_N_3_O_4_ + O_2_ → C_3_H_4_N_3_O_6_
	1	13	9.68–36.60	C_6_H_8_N_7_O_12_ → C_6_H_8_N_7_O_10_ + O_2_
		12	9.65–14.10	C_6_H_8_N_7_O_10_ + O_2_ → C_6_H_8_N_7_O_12_
	0	11	14.38–42.80	C_3_H_4_N_4_O_6_ → C_3_H_4_N_4_O_4_ + O_2_
		11	14.33–46.85	C_3_H_4_N_4_O_4_ + O_2_ → C_3_H_4_N_4_O_6_
9 [001]	−1	57	13.53–58.30	C_3_H_4_N_4_O_7_ → C_3_H_4_N_4_O_5_ + O_2_
		58	13.54–58.28	C_3_H_4_N_4_O_5_ + O_2_ → C_3_H_4_N_4_O_7_
	2	15	28.75–54.33	C_3_H_4_N_4_O_6_ → C_3_H_4_N_4_O_4_ + O_2_
		13	28.78–54.30	C_3_H_4_N_4_O_4_ + O_2_ → C_3_H_4_N_4_O_6_
	−1	6	39.08–42.20	C_6_H_8_N_7_O_10_ → C_6_H_8_N_7_O_8_ + O_2_
		7	39.10–43.18	C_6_H_8_N_7_O_8_ → C_6_H_8_N_7_O_10_ + O_2_
	3	9	46.13–48.15	C_9_H_13_N_11_O_18_ → C_9_H_13_N_11_O_16_ + O_2_
		6	46.73–47.55	C_9_H_13_N_11_O_16_ + O_2_ → C_9_H_13_N_11_O_18_
	2	7	31.90–34.60	C_9_H_12_N_12_O_19_ → O_2_ + C_3_H_4_N_3_O_4_ + C_6_H_8_N_9_O_13_
		5	31.00–34.58	C_6_H_8_N_9_O_13_ + O_2_ + C_3_H_4_N_3_O_4_ → C_9_H_12_N_19_O_19_
	1	5	48.50–49.85	C_9_H_12_N_11_O_17_ → C_9_H_12_N_11_O_15_ + O_2_
		4	48.88–49.90	C_9_H_12_N_11_O_15_ + O_2_ → C_9_H_12_N_11_O_17_

**Table 6:** Reactions of NO_3_ molecule production.

Shock Velocity and Directions (km∙s^−1^)	Net Reaction Frequency	Frequency	Reaction Time (ps)	Reaction
9 [100]	7	24	1.73–27.38	C_3_H_4_N_5_O_8_ → C_3_H_4_N_4_O_5_ + NO_3_
		17	4.95–31.83	C_3_H_4_N_4_O_5_ + NO_3_ → C_3_H_4_N_5_O_8_
	−1	13	6.03–24.68	C_3_H_4_N_4_O_7_ → C_3_H_4_N_3_O_4_ + NO_3_
		14	5.83–19.90	C_3_H_4_N_3_O_4_ + NO_3_ → C_3_H_4_N_4_O_7_
	3	12	3.68–38.43	C_3_H_4_N_5_O_7_ → C_3_H_4_N_4_O_4_ + NO_3_
		9	4.25–30.18	C_3_H_4_N_4_O_4_ + NO_3_ → C_3_H_4_N_5_O_7_
	−2	10	29.03–33.00	C_3_H_3_N_4_O_6_ → C_3_H_3_N_3_O_3_ + NO_3_
		12	29.00–32.98	C_3_H_3_N_3_O_3_ + NO_3_ → C_3_H_3_N_4_O_6_
	2	8	22.43–28.78	C_3_H_4_N_4_O_6_ → C_3_H_4_N_3_O_3_ + NO_3_
		6	25.97–28.85	C_3_H_4_N_3_O_3_ + NO_3_ → C_3_H_4_N_4_O_6_
9 [010]	25	173	2.70–48.00	C_3_H_4_N_5_O_8_ → C_3_H_4_N_4_O_5_ + NO_3_
		148	3.48–47.93	C_3_H_4_N_4_O_5_ + NO_3_ → C_3_H_4_N_5_O_8_
	−6	54	7.58–47.48	C_3_H_4_N_4_O_7_ → C_3_H_4_N_3_O_4_ + NO_3_
		60	7.45–47.78	C_3_H_4_N_3_O_4_ + NO_3_ → C_3_H_4_N_4_O_7_
	−3	34	6.38–49.53	C_3_H_4_N_5_O_9_ → C_3_H_4_N_4_O_6_ + NO_3_
		37	6.35–49.05	C_3_H_4_N_4_O_6_ + NO_3_ → C_3_H_4_N_5_O_9_
9 [001]	14	124	2.23–46.23	C_3_H_4_N_5_O_8_ → C_3_H_4_N_4_O_5_ + NO_3_
		110	2.50–59.85	C_3_H_4_N_4_O_5_ + NO_3_ → C_3_H_4_N_5_O_8_
	2	86	11.70–53.73	C_3_H_4_N_4_O_7_ → C_3_H_4_N_3_O_4_ + NO_3_
		84	12.93–53.75	C_3_H_4_N_3_O_4_ + NO_3_ → C_3_H_4_N_4_O_7_
	1	7	9.43–52.03	C_3_H_4_N_5_O_9_ → C_3_H_4_N_4_O_6_ + NO_3_
		6	9.00–52.00	C_3_H_4_N_4_O_6_ + NO_3_ → C_3_H_4_N_5_O_9_
	−4	8	21.08–48.63	C_6_H_8_N_9_O_15_ → C_6_H_8_N_8_O_12_+ NO_3_
		12	8.70–48.63	C_6_H_8_N_8_O_12_ + NO_3_ → C_6_H_8_N_9_O_15_
	4	6	49.55–52.55	C_6_H_7_N_8_O_13_ → C_6_H_7_N_7_O_10_ + NO_3_
		2	49.65–50.97	C_6_H_7_N_7_O_10_ + NO_3_ → C_6_H_7_N_8_O_13_

**Table 7:** Reactions of HNO_2_ molecule production.

Shock Velocity and Directions (km∙s^−1^)	Net Reaction Frequency	Frequency	Reaction Time (ps)	Reaction
9 [100]	−8	18	12.45–37.68	C_3_H_5_N_5_O_7_ → C_3_H_4_N_4_O_5_+HNO_2_
		26	23.43–37.62	C_3_H_4_N_4_O_5_+HNO_2_ → C_3_H_5_N_5_O_7_
	−4	14	6.33–31.05	C_3_H_5_N_4_O_6_ → C_3_H_4_N_3_O_4_+HNO_2_
		18	6.30–31.10	C_3_H_4_N_3_O_4_+HNO_2_ → C_3_H_5_N_4_O_6_
	−1	6	30.40–34.08	C_3_H_5_N_4_O_8_ → C_3_H_4_N_3_O_6_+HNO_2_
		7	30.45–34.10	C_3_H_4_N_3_O_6_+HNO_2_ → C_3_H_5_N_4_O_8_
	2	5	26.68–29.58	C_3_H_4_N_4_O_7_ → C_3_H_3_N_3_O_5_+HNO_2_
		3	27.18–29.52	C_3_H_3_N_3_O_5_+HNO_2_ → C_3_H_4_N_4_O_7_
	−1	4	17.28–30.05	C_3_H_5_N_5_O_8_ → C_3_H_4_N_4_O_6_+HNO_2_
		5	5.90–19.55	C_3_H_4_N_4_O_6_+HNO_2_ → C_3_H_5_N_5_O_8_
	2	3	4.70–32.88	C_3_H_4_N_4_O_6_ → C_3_H_3_N_3_O_4_+ HNO_2_
		1	26.78–26.78	C_3_H_3_N_3_O_4_+ HNO_2_ → C_3_H_4_N_4_O_6_
9 [010]	1	8	40.03–42.95	C_3_H_4_N_5_O_9_ → C_3_H_3_N_4_O_7_+ HNO_2_
		7	40.05–43.00	C_3_H_3_N_4_O_7_+ HNO_2_ → C_3_H_4_N_5_O_9_
	2	7	52.30–54.85	C_3_H_5_N_5_O_6_ → C_3_H_4_N_4_O_4_+ HNO_2_
		5	52.28–24.83	C_3_H_4_N_4_O_4_+ HNO_2_ → C_3_H_5_N_5_O_6_
	1	6	11.75–54.68	C_3_H_4_N_4_O_6_ → C_3_H_3_N_3_O_4_+ HNO_2_
		5	17.53–55.15	C_3_H_3_N_3_O_4_+ HNO_2_ → C_3_H_4_N_4_O_6_
	−1	3	30.40–53.03	C_3_H_5_N_5_O_7_ → C_3_H_4_N_4_O_5_ +HNO_2_
		4	48.03–53.00	C_3_H_4_N_4_O_5_ +HNO_2_ → C_3_H_5_N_5_O_7_
9 [001]	5	38	2.55–51.28	C_3_H_4_N_4_O_6_ → C_3_H_3_N_3_O_4_+ HNO_2_
		33	3.45–51.28	C_3_H_3_N_3_O_4_+ HNO_2_ → C_3_H_4_N_4_O_6_
	2	32	19.88–58.43	C_3_H_5_N_4_O_6_ → C_3_H_4_N_3_O_4_+ HNO_2_
		30	19.90–58.68	C_3_H_4_N_3_O_4_+ HNO_2_ → C_3_H_5_N_4_O_6_
	−1	6	35.68–56.05	C_3_H_5_N_5_O_7_ → C_3_H_4_N_4_O_5_ +HNO_2_
		7	36.30–56.10	C_3_H_4_N_4_O_5_ +HNO_2_ → C_3_H_5_N_5_O_7_

## Data Availability

The data presented in this study are available in the Supplementary Material.

## Supplementary Materials

The following supporting information can be downloaded at: https://www.mdpi.com/article/10.3390/molecules27185773/s1. Figure S1: Total energy curve of the TNAZ system under different shock wave loading conditions. It can be seen from the figure that the energy curve is continuously rising, indicating that the total energy of the computing system is not conserved. In the slow chemical reaction stage, the nonconservation of energy may be caused by the explicit integration algorithm of the LAMMPS calculation program, Table S1: List of bond order minimum values used to determine molecules, Table S2: Initial shock pressure and stable pressure.
