# Supplementary material for: The Anisotropic Chemical Reaction Mechanism of 1,3,3-trinitroazetidine (TNAZ) under Different Shock Wave Directions by ReaxFF Reactive Molecular Dynamics Simulations
Source: Molecules. 2022 Sep 6;27(18):5773. doi: 10.3390/molecules27185773 (PMC9502667; doi:10.3390/molecules27185773)
Supplement: Supplementary file 1 [file molecules-27-05773-s001.zip › molecules-1886090-supplementary.pdf]

The code is shown as below: (9 km/s [100])

```
units                   real
atom_style   charge
read_restart         tnaz150000.restart
reset_timestep    0
pair_style     reax/c NULL lgvdw yes safezone 5.0 mincap 1000
pair_coeff     * * ffield.reax.lg C H N O
neighbor       2 bin
neigh_modify      every 10 delay 0 check no

fix                     1 all qeq/reax 1 0.0 10.0 1.0e-6 reax/c

fix                     msst all msst x 0.09 q 50.0 mu 0.001 tscale 0.01

fix_modify           msst energy yes
variable             dhug equal f_msst[1]
variable             dray equal f_msst[2]
variable             lgr_vel equal f_msst[3]
variable             lgr_pos equal f_msst[4]

fix              4 all reax/c/bonds 500 tnaz6x.bonds
fix         5 all reax/c/species 10 10 100 species6x.tnaz element C H N O

timestep 0.05

thermo                  10
thermo_style        custom step temp press vol density pe ke lz pzz etotal v_dhug v_dray v_lgr_vel v_lgr_pos
f_msst cella cellb cellc cellalpha cellbeta cellgamma

dump                    1 all custom 500 tnaz6x.lammpstrj id type xs ys zs vx vy vz
dump                    2 all cfg 1000 dump.snap.*.cfg mass type xsu ysu zsu id
dump_modify          2 element C H N O
restart          10000 tmp*.restart
run                     2000000

write_restart      tmp*.restart
```

Table S1. List of Bond Order Minimum Values Used to Determine Molecules

Atom type	Atom type	Bond order
C	C	0.30
C	H	0.40
C	N	0.30
C	O	0.65
H	H	0.55
H	N	0.40
H	O	0.40
N	N	0.30
N	O	0.40
O	O	0.65

Table S2. Initial shock pressure and stable pressure

Velocity/km·s^{-1}	Initial pressure value/GPa	Corresponding Time/ps	Stable pressure value/GPa	Corresponding Time/ps
8 [100]	33.55	12.56	--	--
8 [010]	33.31	12.76	--	--
8 [001]	33.60	4.67	--	--
9 [100]	46.97	11.83	60.66	46.84
9 [010]	46.62	11.10	59.10	63.30
9 [001]	46.54	7.25	59.50	75.53
10 [100]	64.14	2.98	84.77	12.71
10 [010]	62.47	3.74	83.30	16.23
10 [001]	63.02	5.38	83.86	16.75

Figure S1 is the total energy curve of the TNAZ system under different shock wave loading conditions. It can be seen from the figure that the energy curve is continuously rising, indicating that the total energy of the computing system is not conserved. In the slow-chemical-reaction stage, the non-conservation of energy may be caused by the explicit integration algorithm of the Lammps calculation program.

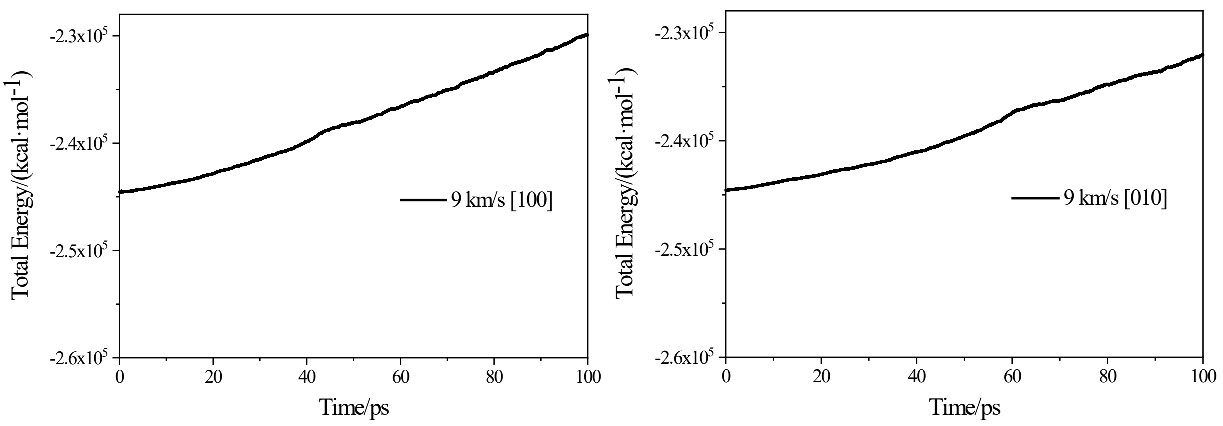

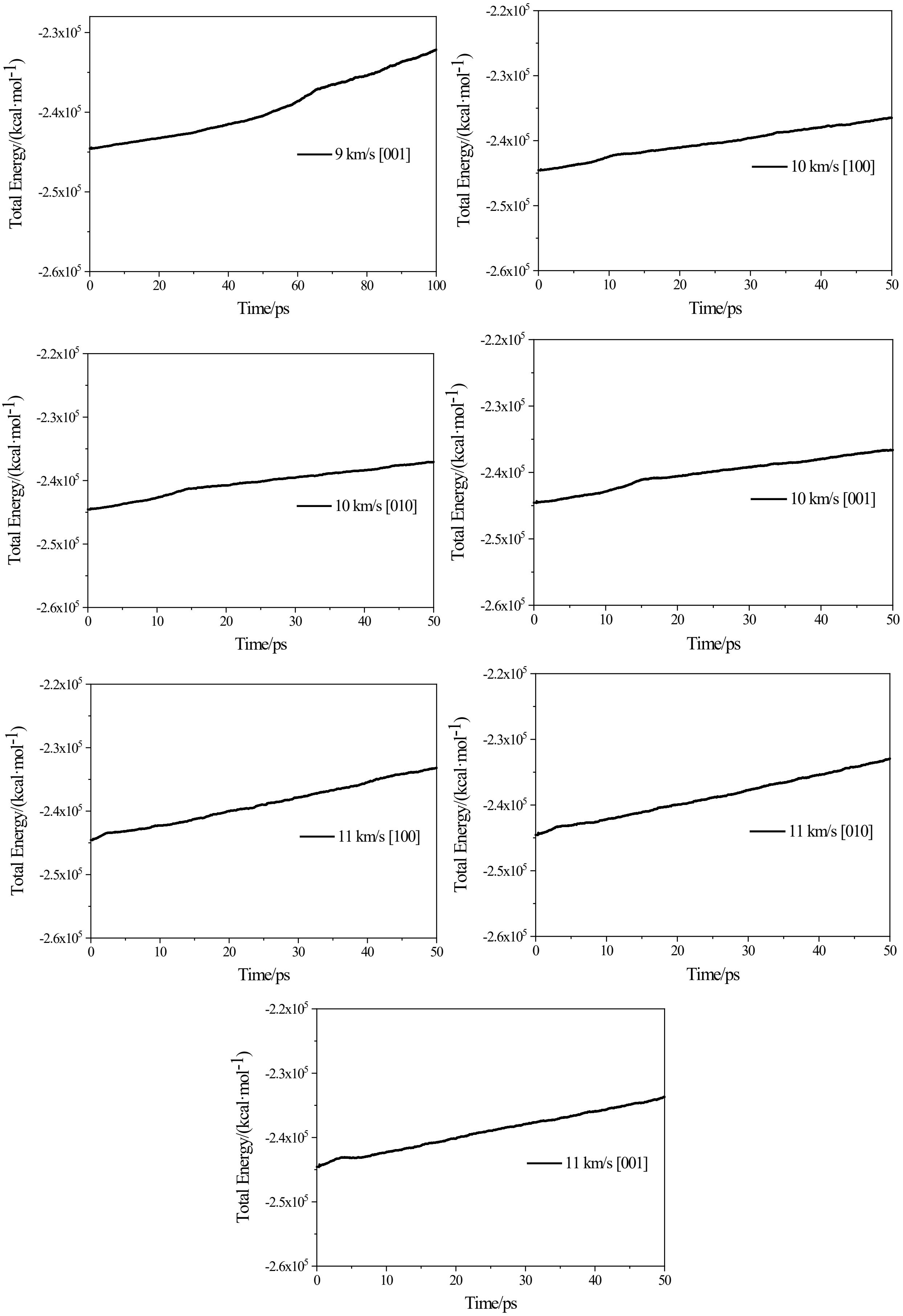

Figure S1 Total energy of TNAZ system
